# Supplementary material for: Metabolites of traffic-related volatile organic compounds in age-related macular degeneration
Source: PeerJ. 2025 Dec 3;13:e20405. doi: 10.7717/peerj.20405 (PMC12681231; doi:10.7717/peerj.20405)
Supplement: Supplemental Information 1 [file peerj-13-20405-s001.pdf]

The CONSORT-PRO Reporting Guidance Checklist

Section/Topic	CONSORT-PRO Item	Recommended Content	Page Addressed
**Title and Abstract**			
	P1b	The PRO should be identified in the abstract as a primary or secondary outcome.	2
**Introduction**			
Background and objectives	2a	The scientific background and explanation of rationale of PRO assessment should be included.	3
	P2b	The PRO hypothesis should be stated, and relevant domains identified, if applicable.	3
**Methods**			
Participants	4a	PRO-specific criteria are required only if PROs were used for eligibility or stratification.	4
Outcomes	P6a	Evidence of PRO instrument validity and reliability should be provided or cited if available including the person completing the PRO and methods of data collection (paper, telephone, electronic).	4-5
Sample size	7a	Sample size determination is required only if PRO is a primary study outcome.	5-6
**Randomization**			
Statistical methods	P12a	Statistical approaches for dealing with missing data are explicitly stated.	6-7
**Results**			
Participant flow	13a	The number of PRO outcome data at baseline and at subsequent time points should be transparent.	7
Baseline data	15	PRO data in the table showing baseline demographic and clinical characteristics for each group should be included.	7
Numbers analyzed	16	For each group, the number of participants (denominator) included in each analysis and whether the analysis was by original assigned groups) is required for PRO results.	7-10
Outcomes and estimation	17a	The estimated effect size and its precision such as 95% confidence interval should be presented for multidimensional PROs from each domain and time point.	7-10
Ancillary analyses	18	Results of any other PRO analyses performed, including subgroup analyses and adjusted analyses, distinguishing pre-specified from exploratory should be presented, where relevant.	7-10
**Discussion**			
Limitation	P20/21	PRO-specific limitations and implications for generalizability and clinical practice should be presented.	12
Interpretation	22	PRO data should be interpreted in relation to clinical outcomes including survival data, where relevant.	12

Calvert M, Blazeby J, Altman DG, et al. Reporting of patient-reported outcomes in randomized trials: the CONSORT PRO extension. *JAMA*. 2013;309(8):814-822. doi:10.1001/jama.2013.879

Note: The CONSORT-PRO Extension should be used with the CONSORT 2010 Statement and any other relevant CONSORT Extensions, found at consort-statement.org
