# Supplementary material for: Metabolites of traffic-related volatile organic compounds in age-related macular degeneration
Source: PeerJ. 2025 Dec 3;13:e20405. doi: 10.7717/peerj.20405 (PMC12681231; doi:10.7717/peerj.20405)
Supplement: Supplemental Information 3 [file peerj-13-20405-s003.docx]

. **研究実施計画書**

1. 課題名：眼疾患における生理活性物質および代謝産物の解析
2. 研究の意義（背景）と目的

我々はこれまでに涙液、眼内液（前房水と硝子体液）、血液、尿中のVEGF, IL-6, PEDFなどの生理活性物質・代謝産物と眼疾患の病態との関係性について研究をしてきた。本研究では、対象疾患を広げて、眼疾患の病態と生理活性物質・代謝産物との関連性について調査することを目的とする。

1. 研究の方法：

研究の対象：眼科疾患を有し、当院眼科外来通院患者を対象とする。

対象の除外基準：なし

血液・尿の採取：眼科外来にて血液5mlおよび尿のサンプル5mlを、研究目的で採取する。

涙液、眼内液採取：当院眼科外来通院患者のうち、手術（硝子体手術、白内障手術、緑内障手術時）を行う対象者において、手術時に廃棄する眼内液（前房水）を0.2mlを研究目的にて利用する。この採取量は日常診療時に眼内液を外来にて採取するのと同じ量である。

血液・尿・眼内液の測定項目はVEGF関連因子、ビタミンB複合体、分子鎖アミノ酸、クエン酸、糖代謝、脂肪酸代謝、腸代謝、肝臓解毒インディケータ、神経伝達物質、TCAサイクル、自動車源物質、塗料因子物質、パーソナルケア製品源物質、フタル酸類、プラスチック源物質、水源物質を測定する。検体は検査に全量使用するため、検査後に残らない。したがって、検査後に使用したすべての検体を廃棄し、保存は行わない。各因子の解析にはELISA法を用いる。

1. 目標対象者数：

各眼疾患20名

症例数の算定根拠：過去の我々の同様の研究（Yokosako, Mimura et al. Open Ophthalmol J. 2014）では、加齢黄斑変性の患者17例で解析が可能であった。過去の研究では加齢黄斑変性患者において、既知の健常者の標準基準値を比較した場合、糖代謝とTCAサイクルにおける生理活性物質の濃度が、いずれも統計学的な有意差P<0.05をもって増加していることが証明できた。従って、本研究においては、過去の研究と同様に、既知の健常者の標準基準値と比較する方法をとるため、いずれの生理活性物質の濃度の比較においても、脱落例を含め、各疾患において、20例をエントリーできれば、十分、統計解析が可能と考えられる。

1. 研究組織（○本学における実施責任者　◎本学における情報管理責任者）：

研究責任者

溝田　淳　　　眼科学講座　　　　主任教授

参加者

林　孝雄　　　眼科学講座　　　　兼担准教授

◎〇三村達哉　　　眼科学講座　　　　准教授

渡邊恵美子　　眼科学講座　　　　病院准教授

太根ゆさ　　　眼科学講座　　　　助教

水野嘉信　　　眼科学講座　　　　助教

松本浩一　　　眼科学講座　　　　助手

寺内　岳　　　眼科学講座　　　　助手

川嶋真　　　　眼科学講座　　　　臨床助手

矢倉和磨　　　眼科学講座　　　　臨床助手

越智正登　　　眼科学講座　　　　修練医

浜野茂樹　　　眼科学講座　　　　臨床助手

酒井浩之　　　眼科学講座　　　　臨床助手

南波久貴　　　眼科学講座　　　　臨床助手

生方翔子　　　医学部附属病院　　シニアレジデント

北川達士　　　医学部附属病院　　シニアレジデント

吉津和真　　　医学部附属病院　　シニアレジデント

1. 研究期間：

倫理委員会承認後～2030年3月31日まで。

1. 研究における倫理的配慮：

本研究は、「ヘルシンキ宣言に基づく倫理的原則」および「人を対象とする医学系研究に関する倫理指針」に従い、本計画書を遵守して実施する。

1）インフォームド・コンセント

研究担当者は、倫理委員会で承認の得られた説明文書、同意書を被験者に渡し、文書および口頭による十分な説明を行い、被験者の自由意思による同意を文書で取得する。

研究担当者は、被験者の同意に影響を及ぼす情報が得られたときや、被験者の同意に影響を及ぼすような実施計画等の変更が行われるときは、速やかに被験者に情報提供し、研究に参加するか否かについて被験者の意思を予め確認するとともに、事前に倫理委員会の承認を得て説明文書、同意書等の改訂を行い、被験者の再同意を得ることとする。

説明文書、同意書には、以下の内容を含むものとする。

①研究への参加は任意であること、同意しなくても不利益を受けないこと、同意は撤回できること

②研究の意義（背景）、目的（眼加齢疾患において生理活性物質・代謝産物との関連性を調べること）、対象（20歳以上の眼加齢疾患を有する患者）、方法（手術中の廃液となる眼内前房水と硝子体液、および、尿、血液を採取して、組成を調べる）、実施期間（約2年間）、予定被験者数（各疾患30例）

③研究に参加することにより期待される利益、起こりえる不利益（眼の軽微な侵襲）

④個人情報を含めた試料等の取扱い、保存期間と廃棄方法、研究方法等の閲覧

⑤研究成果の発表および特許が発生した場合の取扱い

⑥研究に係る被験者の費用負担、研究資金源と利益相反

⑦研究の組織体制、研究に関する問い合わせ、苦情等の相談窓口（連絡先）

⑧被験者に健康被害が発生した場合の対応と補償の有無

**連絡方法・連絡者・連絡先**

質問、 資料閲覧、 同意 撤回 など 参加者が随時、連絡したい場合。

【連絡方法】外来で直接担当医に連絡頂くか、事務局に電話で確認頂く。

【連絡者】担当医を介して研究責任者に連絡頂くか、研究事務局（研究責任者）に直接連絡頂く。

【連絡先】住　所：〒173-8605 東京都板橋区加賀2-11-1

帝京大学医学部附属病院眼科　准教授　三村達哉

電話：03-3964-1211　（32626）

2）情報（研究に用いられる情報に係る資料を含む）の保管及び廃棄の方法

全ての患者データは研究開始時に名前とIDの対応表を別のエクセルファイルに保管することにより、解析に用いるデータを匿名化にし、情報管理責任者（三村達哉）が責任をもって管理する。デジタルデータについては、起動後のパスワードが設定された帝京大学眼科医局内に設置された専用のパソコンで保管する。患者名、ID番号は用いず、全てナンバリングにより保管し、ナンバリングデータの照合はパスワードによってファイルを管理する。また、個人が同定されないようにデータを集計し、患者に関する個人情報は公表されない。

本研究から得られた情報が、研究対象者等から同意を受ける時点では特定されない将来の研究のために用いられる可能性、又は他の研究機関に提供する可能性がある。

被験者が研究参加の意思表示を撤回した場合、講座内で保管した診療情報等から抽出したデータ、アンケート等の資料はシュレッダーにて裁断し、デジタルデータは専用のパソコンの内蔵ハードディスクから、専用のデータ消去ソフト（ドライブイレーサーなど）を用いて完全に削除する。

研究等の実施に係わる重要な文書（申請書類の控え、理事長及び病院長からの通知文書、各種申請書・報告書の控、同意書、その他データの信頼性を保証するのに必要な書類または記録等）およびデータは、情報管理責任者により研究の終了後に倫理委員会事務局に提出され、10年が経過した日までの間、帝京大学臨床研究センター（TARC）にて保存される。TARCに保管されたデータはTARCがTARC記録保管手順書ならびに臨床研究における記録保管に関する標準業務手順書に従い、個人情報に注意してシュレッダーで裁断した後に廃棄する。

**問い合わせ・苦情等に対する連絡先**

【連絡先】住　所：〒173-8605 東京都板橋区加賀2-11-1

帝京大学医学部附属病院眼科　三村達哉

電話：03-3964-1211　（32626）

3）試料の保管及び廃棄の方法

該当なし

1. 研究に参加することにより起こりうる危険並びに必然的に伴う心身に対する不快な状態（有害事象）：重篤な有害事象発生時には、手順書に従って病院長と倫理委員会事務局に速やかに報告する。検体採取目的のみで前房水を採取することはなく、眼手術時に廃棄する眼内液を利用する。手術による侵襲および採血による侵襲は以下の通りである。

1．手術時に廃棄する眼内液を採取するため、眼内液を採取する行為自体には侵襲を伴わない。手術により、眼表面の損傷などの有害事象が発生した場合には、角膜保護点眼薬を用いて治療する。

2．採血室にて採血時に、皮下出血が生じた場合には圧迫止血を行い、採血後に末梢神経障害が生じた場合には、消炎鎮痛薬とともに、神経障害性痛に対する薬剤を使用する。

1. 研究に参加することにより生じる利益および試験期間中にかかる対象者の医療費：

利益：特になし

医療費：本研究は、研究目的（血液・尿の採取および眼内液採取）で行われるため、保険診療以外に医療費は発生しない。各因子の測定は眼科学講座研究費により測定するため、研究に参加することによる患者の費用負担は発生しない。検体は白内障手術、硝子体手術、緑内障手術時に廃棄する眼内液を使用するため、眼内液を採取する行為自体に侵襲を伴うことはない。通常の手術時に眼を損傷した場合には、研究目的ではなく、手術そのものに対する侵襲および合併症であり、その治療費用は保険診療下で患者の自己負担で治療が行われる。

1. 研究の中止の条件とその対応：

研究の中止の条件：研究担当者は、以下の事項に該当する場合は、研究中止を検討する。

①被験者の組み入れが困難で、予定症例数に達することが困難であると判断されたとき。

②予定症例数または予定期間に達する前に、研究の目的が達成されたとき。

③倫理委員会により、実施計画等の変更の指示があり、これを受入れることが困難と判断されたとき。

対応：研究責任者は、倫理委員会により中止の勧告あるいは指示があった場合は、研究を中止する。また、研究の中止または中断を決定した時は、速やかに理事長及び病院長にその理由とともに文書で報告する。また、倫理委員会ならびに研究対象者に、文書で報告をする。情報、資料は5年間保管後に7(3)の項に従って、廃棄する。

1. モニタリング及び監査：

該当なし

1. 研究実施後の研究対象者への対応：

研究責任者および担当者は、当該研究実施後においても、研究対象者が当該研究の結果により得られた最善の医療（予防、診断及び治療）を受けることができるように努める。なお、「研究対象者への研究実施後」とは、研究計画書に記載された研究期間が満了したときではなく、個々の研究対象者に対して通常の診療を終了した後を指す。

1. 研究成果の取りまとめとその扱い：

本研究の研究成果の公表の可否および公表する内容は、研究責任者が選任する講座内の研究委員会が決定する。研究担当者は、本研究の結果を、個人が同定されないよう集計値として結果を取りまとめて解析し、成果を関連学会等において発表の後、論文として紙面にて公表する。成果の発表には匿名化されたデータを使用するため、個人が特定されることはない。本研究の成果物としての知的財産権については、帝京大学医学部附属病院眼科に帰属する。

1. 研究資金源：

診療の範囲内で行う検査であるため、研究費は必要としない。学会での発表、紙面上での公表等で資金が必要となる場合には、研究責任者、担当者が所属する診療科の研究費（文部科研費・基盤研究（　C　）16K11332で実施する。

1. 利益相反：

本研究の研究担当者は、「利益相反マネジメント規程」に従って、帝京大学板橋キャンパス利益相反管理委員会に必要事項を申告し、その審査と承認を得るものとする。研究によって得られる経済的利益（企業団体等からの寄附等。特許よるものを除く）あるいは、研究に関連する企業団体等からの研究対象機器等の無償貸与、購入に関する利益相反の問題はない。

1. 倫理委員会への経過および最終報告予定：

実際に開始した時期、実施状況（症例数）、倫理的配慮の状況、不利益・有害事象の発生状況、研究成果などについて、年１回中間報告書を、提出する。研究の終了時には、研究責任者は速やかに最終報告書を倫理委員会に提出する。重篤な有害事象発生時には手順書に従って病院長と倫理委員会事務局に速やかに報告する。

研究開始時に、UMIN臨床試験登録システムに登録し、データベースについては、国立大学附属病院長会議、一般財団法人日本医薬情報センター又は社団法人日本医師会が設置している公開データベースに登録する。

1. 添付資料一覧：

特になし
