# Supplementary material for: Metabolites of traffic-related volatile organic compounds in age-related macular degeneration
Source: PeerJ. 2025 Dec 3;13:e20405. doi: 10.7717/peerj.20405 (PMC12681231; doi:10.7717/peerj.20405)
Supplement: Supplemental Information 4 [file peerj-13-20405-s004.docx]

. **Research Protocols**

1. Title: Analysis of bioactive substances and metabolites in ocular diseases
2. Significance (Background) and Purpose of the Research

We have studied the relationship between bioactive substances and metabolites such as VEGF, IL-6, and PEDF in tears, intraocular fluid (anterior aqueous humor and vitreous fluid), blood, and urine and the pathophysiology of eye diseases. The purpose of this study is to investigate the relationship between the pathophysiology of eye diseases and bioactive substances and metabolites.

1. Methods of study:

Subject of the study: Patients with ophthalmic diseases who visit our hospital's ophthalmology outpatient clinic.

Exclusion criteria: None

Blood and urine collection: 5 ml of blood and 5 ml of urine samples will be collected at the ophthalmology outpatient clinic for research purposes.

Tear fluid and intraocular fluid collection: In patients who are outpatient in the ophthalmology department of our hospital and are undergoing surgery (vitreous surgery, cataract surgery, glaucoma surgery), 0.2 ml of intraocular fluid (anterior aqueous humor) that is discarded at the time of surgery is used for research purposes. This collection amount is the same as that of intraocular fluid collected on an outpatient basis during routine medical care.

Measurement items of blood, urine, and intraocular fluids measure VEGF-related factors, vitamin B complex, molecular chain amino acids, citric acid, glucose metabolism, fatty acid metabolism, intestinal metabolism, liver detoxification indicators, neurotransmitters, TCA cycles, automotive source substances, paint factor substances, personal care product source substances, phthalates, plastic source substances, and water source substances. Since the entire amount of the specimen is used for testing, it does not remain after the test. Therefore, all specimens used after the test are discarded and not stored. The ELISA method is used for the analysis of each factor.

1. Target number of targets:

20 patients with each eye disease

Basis for calculating the number of cases: Similar studies of our past (Yokosako, M imuraet al. Open Ophthalmol J. 2014Analysis was possible in 17 patients with age-related macular degeneration. In previous studies, patients with age-related macular degeneration were able to demonstrate an increase in the concentration of bioactive substances in glucose metabolism and the TCA cycle with a statistically significant difference of P<0.05 when comparing the standard reference values of known healthy subjects. Therefore, in this study, as in past studies, we will use a method to compare the standard reference values of known healthy subjects, so if we can enter 20 cases for each disease, including those who have dropped out, in the comparison of the concentrations of biologically active substances, it is considered that statistical analysis is sufficient.

1. Research organization (○ Person in charge of implementation at the University ◎ Person in charge of information management at the University):

Principal Investigator

Jun Mizota, Senior Professor, Department of Ophthalmology

participant

Lin Xiaoxiong is an associate professor in ophthalmology

◎〇Tatsuya Mimura, Department of Ophthalmology, Associate Professor

Emiko Watanabe, Department of Ophthalmology, Associate Professor, Hospital

Yusa Ohne, Assistant Professor, Department of Ophthalmology

Yoshinobu Mizuno, Assistant Professor, Department of Ophthalmology

Koichi Matsumoto, Assistant Professor, Department of Ophthalmology

Terauchi Yue ophthalmology lecture assistant

Makoto Kawashima, Clinical Assistant, Department of Ophthalmology

Kazuma Yagura, Department of Ophthalmology, Clinical Assistant

Masato Ochi, Department of Ophthalmology, Trainee Physician

Shigeki Hamano, Department of Ophthalmology, Clinical Assistant

Hiroyuki Sakai, Department of Ophthalmology, Clinical Assistant

Hisaki Nanba, Department of Ophthalmology, Clinical Assistant

Shoko Ikukata, Senior Resident, University Hospital

Tatsushi Kitagawa, Senior Resident, University Hospital

Kazuma Yoshitsu, Senior Resident, University Hospital

1. Study Period:

After approval by the Ethics Committee ~ until March 31, 2030.

1. Ethical considerations in research:

This research will be conducted in accordance with the "Ethical Principles Based on the Declaration of Helsinki" and the "Ethical Guidelines for Medical Research on Human Subjects" and in compliance with this plan.

1) Informed consent

The investigator will provide the subject with an explanatory document and consent form approved by the ethics committee, provide sufficient written and oral explanations, and obtain the subject's free and voluntary consent in writing.

When information that affects the consent of the subject is obtained, or when a change is made to the implementation plan, etc., that affects the consent of the subject, the person in charge of the research shall promptly provide the information to the subject, confirm the subject's intention in advance whether or not to participate in the study, and obtain the consent of the subject by revising the explanatory document, consent form, etc., with the approval of the Ethics Committee in advance.

Explanatory documents and consent forms shall include the following contents:

(1) Participation in the research is voluntary, and consent can be withdrawn.

(2) Significance (background), purpose (to investigate the relationship between bioactive substances and metabolites in ocular age-related diseases), target (patients aged 20 years or older with ocular age-related diseases), method (intraocular anterior aqueous humor and vitreous fluid, which are waste fluids during surgery, urine, and blood are collected to investigate the composition), implementation period (about 2 years), planned number of subjects (30 subjects for each disease)

(3) Expected benefits and possible disadvantages of participating in the study (minor invasion of the eye)

(4) Handling of samples, etc., including personal information, storage period, disposal method, inspection of research methods, etc.

(5) Announcement of research results and handling of patents

(6) Subjects' expenses, research funding sources, and conflicts of interest

(7) Organizational structure of the research, consultation desk for inquiries and complaints regarding the research (contact information)

(8) Response in the event of health damage to the subject and whether or not there is compensation

**Contact method, contact person, contact information**

Questions, material viewing, consent withdrawal, etc. if the participant wishes to contact us at any time.

【How to contact】Please contact your doctor directly at the outpatient clinic or call the secretariat to confirm.

Contact the Principal Investigator through the Principal Investigator or directly with the Principal Investigator (Principal Investigator).

Contact: 〒173-8605 2-11-1 Kaga, Itabashi-ku, Tokyo

Associate Professor, Department of Ophthalmology, Teikyo University Hospital, Tatsuya Mimura

Tel: 03-3964-1211 (32626)

2) Methods of storage and disposal of information (including materials related to information used in research)

All patient data is anonymized by storing a name-ID correspondence table in a separate Excel file at the start of the study, and the information manager (Tatsuya Mimura) is responsible for managing it. Digital data is stored on a dedicated computer installed in the Teikyo University Ophthalmologist's Office, which has a password after startup. Patient names and ID numbers are not used, and all files are stored by numbering, and the file is managed by password for collating numbering data. In addition, data is aggregated so that individuals are not identified, and personal information about patients is not made public.

The information obtained from this study may be used for future research that is not specified at the time of obtaining consent from the research subject, etc., or may be provided to other research institutions.

If the subject withdraws his or her intention to participate in the study, the data extracted from the medical information, questionnaires, and other materials stored in the course will be shredded, and the digital data will be completely deleted from the internal hard disk of the dedicated computer using dedicated data erasing software (such as a drive eraser).

Important documents related to the conduct of research (copies of application documents, notification documents from the president and hospital director, receipts of various applications and reports, consent forms, and other documents or records necessary to guarantee the reliability of the data) and data will be submitted to the Ethics Committee Secretariat after the completion of the research by the information manager, and will be preserved at the Teikyo University Clinical Research Center (TARC) until the date 10 years have elapsed. Data stored in TARC shall be disposed of after TARC has shredded personal information in accordance with TARC Records Retention Procedures and Standard Operating Procedures for Records Keeping in Clinical Research.

**Contact information for inquiries, complaints, etc.**

Contact: 〒173-8605 2-11-1 Kaga, Itabashi-ku, Tokyo

Department of Ophthalmology, Teikyo University Hospital, Tatsuya Mimura

Tel: 03-3964-1211 (32626)

3) Method of storage and disposal of samples

Not applicable

1. Possible hazards and inevitable physical and mental discomfort (adverse events) due to participation in the study: In the event of a serious adverse event, promptly report it to the hospital director and the Ethics Committee Secretariat in accordance with the procedure manual. The anterior chamber fluid is not collected only for the purpose of collecting specimens, but the intraocular fluid that is discarded at the time of eye surgery is used. Surgical invasiveness and blood sampling are as follows.

1. The act of collecting intraocular fluid itself is not invasive because it collects intraocular fluid to be discarded at the time of surgery. If adverse events such as damage to the ocular surface occur due to surgery, treatment is performed with corneal protective eye drops.

2. If subcutaneous hemorrhage occurs at the time of blood collection in the blood collection room, compression hemostasis is performed, and if peripheral neuropathy occurs after blood collection, a drug for neuropathic pain is used together with an anti-inflammatory analgesic.

1. Benefits arising from participation in the study and the subject's medical expenses incurred during the study period:

Profit: None

Medical expenses: Since this study is conducted for research purposes (blood and urine collection and intraocular fluid collection), medical expenses will not be incurred other than insurance treatment. Since the measurement of each factor is measured by the research fund of the Department of Ophthalmology, there is no cost burden for the patient by participating in the study. Since the specimen uses intraocular fluid that is discarded during cataract surgery, vitreous surgery, and glaucoma surgery, the act of collecting intraocular fluid itself is not invasive. In the case of eye damage during normal surgery, it is not for research purposes, but for invasiveness and complications of the surgery itself, and the cost of treatment is paid by the patient under insurance.

1. Conditions for discontinuation of the study and their correspondence:

Conditions for discontinuation of the study: The principal investigator will consider discontinuing the study if any of the following applies:

(1) When it is judged that it is difficult to enroll subjects and it is difficult to reach the planned number of cases.

(2) When the purpose of the study is achieved before the planned number of cases or the planned period is reached.

(3) When the Ethics Committee instructs the Ethics Committee to change the implementation plan, etc., and it is judged that it is difficult to accept the instructions.

Response: The principal investigator will discontinue the research if the Ethics Committee recommends or instructs the research to discontinue. In addition, when a decision is made to discontinue or suspend the research, the reason shall be promptly reported in writing to the president and the director of the hospital. In addition, a written report shall be made to the Ethics Committee and the research subjects. Information and materials shall be stored for five years and then disposed of in accordance with paragraph 7(3).

1. Monitoring & Auditing:

Not applicable

1. Dealing with study subjects after conducting the study:

The principal investigator and the person in charge shall endeavor to ensure that the research subject receives the best medical care (prevention, diagnosis, and treatment) obtained as a result of the research even after the research is conducted. The term "after the conduct of the study on the research subjects" does not refer to the expiration of the research period stated in the research protocol, but to the completion of regular medical treatment for individual research subjects.

1. Compilation of research results and their treatment:

The decision on whether or not to publish the results of this research and the content to be made public will be decided by the research committee in the department appointed by the principal investigator. The person in charge of the research will compile and analyze the results of this study as aggregate values so that individuals are not identified, and publish the results as a paper after presenting the results at related academic conferences. Anonymized data is used to present the results, so individuals are not identified. The intellectual property rights as a result of this research belong to the Department of Ophthalmology, Teikyo University Hospital.

1. Research Funding Source:

Since the test is performed within the scope of medical care, no research expenses are required. If funds are required for presentations at academic conferences or publications in the paper, the research funds of the medical department to which the principal investigator and the person in charge belong (Grants-in-Aid for Scientific Research (C) 16K11332 will be used.

1. Conflict of Interest:

In accordance with the Conflict of Interest Management Regulations, the person in charge of this research shall submit the necessary information to the Conflict of Interest Management Committee of Teikyo University's Itabashi Campus, and obtain its examination and approval. Economic benefits obtained from research (donations from corporate organizations, etc.) There is no conflict of interest in the free loan or purchase of research equipment from corporate organizations related to research.

1. Progress and final report to the Ethics Committee:

An interim report is submitted once a year on the actual timing of the start, the status of implementation (number of cases), the status of ethical considerations, the occurrence of adverse and adverse events, and the results of the research. At the conclusion of the study, the principal investigator promptly submits a final report to the Ethics Committee. In the event of a serious adverse event, promptly report it to the hospital director and the Ethics Committee Secretariat in accordance with the procedure manual.

At the start of the study, the patient will be registered in the UMIN clinical trial registry system, and the database will be registered in a public database established by the National University Hospital Directors' Conference, the Japan Medical Information Center, or the Japan Medical Association.

1. List of attachments:

None
